# Clustering of *Drosophila melanogaster* Immune Genes in Interplay with Recombination Rate

**DOI:** 10.1371/journal.pone.0002835

**Published:** 2008-07-30

**Authors:** K. Mathias Wegner

**Affiliations:** Institute for Integrative Biology, Experimental Ecology, ETH Zürich, Zürich, Switzerland; Washington University School of Medicine in St. Louis, United States of America

## Abstract

**Background:**

Gene order in eukaryotic chromosomes is not random and has been linked to coordination of gene expression, chromatin structure and also recombination rate. The evolution of recombination rate is especially relevant for genes involved in immunity because host-parasite co-evolution could select for increased recombination rate (Red Queen hypothesis). To identify patterns left by the intimate interaction between hosts and parasites, I analysed the genomic parameters of the immune genes from 24 gene families/groups of *Drosophila melanogaster*.

**Principal Findings:**

Immune genes that directly interact with the pathogen (i.e. recognition and effector genes) clustered in regions of higher recombination rates. Out of these, clustered effector genes were transcribed fastest indicating that transcriptional control might be one major cause for cluster formation. The relative position of clusters to each other, on the other hand, cannot be explained by transcriptional control *per se*. *Drosophila* immune genes that show epistatic interactions can be found at an average distance of 15.44±2.98 cM, which is considerably closer than genes that do not interact (30.64±1.95 cM).

**Conclusions:**

Epistatically interacting genes rarely belong to the same cluster, which supports recent models of optimal recombination rates between interacting genes in antagonistic host-parasite co-evolution. These patterns suggest that formation of local clusters might be a result of transcriptional control, but that in the condensed genome of *D. melanogaster* relative position of these clusters may be a result of selection for optimal rather than maximal recombination rates between these clusters.

## Introduction

The sequence of genes in eukaryotic genomes is not random [1]. Several mechanisms could potentially account for the observed patterns, some of which are not a direct result from selection, while others bear signs of selection favouring clustered genes. In the *C. elegans* genome, for example, a major proportion of genes occurs in clusters [2]. This pattern is most likely a consequence of gene duplications placing homologous copies in close vicinity to each other rather than a direct consequence of selection.

Apart from initially neutral processes like gene duplication, several other mechanisms of selective importance could generate clustering of genes. Recent studies focussed on transcriptional regulation as co-regulated genes can be transcribed together more efficiently when they are in close proximity, because condensed chromatin has only to be uncoiled in a few places [3]. Essential genes for example cluster in regions with open chromatin structure to reduce noise in expression patterns [4]. Selection on transcription levels favouring local concentration is not restricted to essential genes but can be extended to other genes [5]–[7], which are often functionally related [8], [9] and also share chromatin organisation [10]. As a consequence, genes located in functionally related clusters should be transcribed faster and at more similar trancription levels than non-clustered genes.

Another evolutionary force that could potentially form gene clusters is selection for linkage disequilibria [11]. This kind of selection is believed to be rather weak, because it involves polymorphism and epistasis between clustered genes. However, if epistasis is synergistic a concentration of these in the same chromosomal region as a so-called “supergene” would be selectively favoured [12] and has been observed for genes controlling colour patterns involved in butterfly mimicry [13].

One group of genes for which the assumptions of epistasis and polymorphism hold are genes involved in the immune response [14], [15]. Detailed analysis revealed that a large proportion of *Drosophila* immune genes interact epistatically with each other [16], [17] and that some *Drosophila* immune receptors can display high levels of linkage disequilibrium [18]. If these patterns are a result of host-parasite co-evolution, the Red Queen hypothesis of antagonistic co-evolution [19], [20] would predict that the sign of epistasis should frequently switch resulting in time lagged fluctuations of linkage disequilibria, which in turn would select for increased recombination rates [21]–[23]. Such fluctuating selection regimes can also be expected to leave their footprint in the genome. If selection maximizes recombination rate between epistatically interacting genes, these genes should be found on different chromosomes resulting in the maximal recombination rate of 50%. In this case immune genes should be evenly spread out across the genome and clustering should be minimal. Recent theoretical considerations came however to the conclusion that recombination rate between loci subjected to antagonistic host-parasite co-evolution should have an optimal rather than maximal recombination rates [24], which would support concentration of immune genes at least on chromosomes. If however, complex epistatic interactions between several genes on one chromosome exist [16], [17] selection for optimal recombination rates would also favour clustering by joining non-interacting genes into one cluster while the interacting partners should be found in other clusters.

To see whether there is a connection between genomic distribution of immune genes and recombination rate, I analysed spatial and recombinatorial patterns of the immunome (i.e. the genes of the immune system) of the well described and functionally characterised genome of *Drosophila melanogaster*. While immune genes most likely do not represent a special case in terms of clustering, they constitute a unique data set that actually allows to evaluate different hypotheses forming genome architecture because extensive data describing the transcriptional response to pathogen infection [25] as well as epistatic interactions of genetic polymorphism between these genes [16], [17]. Specifically, I tried to discriminate between patterns of neutral evolution (i.e. gene duplication) and selection for spatial immunome organisation addressing transcriptional regulation and coevolution in particular. If clustering is mainly a consequence of local gene duplications I would expect to find low gene family diversity in clusters and a positive relationship between recombination rate and cluster size [2]. If selection on transcriptional profiles is strong [4], I would expect an elevated within-cluster co-regulation to facilitate a fast immune response. Transcriptional regulation can however only explain cluster formation of single clusters. The position of gene clusters along a chromosome might be non-random as well and selection for changes in linkage disequilibria as a consequence of antagonistic host-parasite co-evolution could potentially explain such non-random order of clusters. Following theoretical predictions [24], I would predict to find intermediate recombination rates between epistatically interacting genes as opposed to genes that show no interaction. Such a pattern could not only have profound impacts on cluster formation itself, but could also explain genome organisation on the next higher level of the chromosome.

## Results

### Between and within chromosome clustering

**Figure 1:**
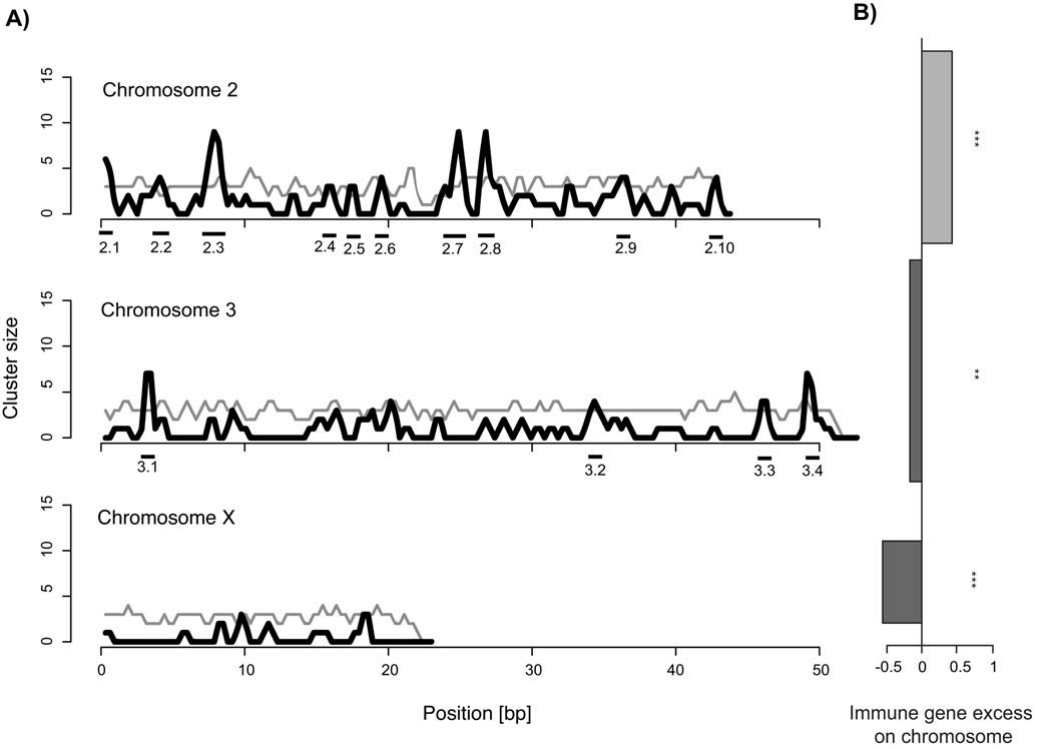
Clustering of immune genes in the *Drosophila melanogaster* genome. A) Clusters of immune genes within chromosomes. The black line shows the observed density of immune genes in a 2 cM sliding window (step size 1 cM). The grey line shows the 95% quantile densities in corresponding windows from 1′000 randomly distributed sets of immune genes per chromosome. Significant clusters were defined for those steps where the observed density exceeded the maximum random density and are indicated as black bars underneath the x-axis. B) Excess of immune genes on corresponding chromosomes. Excess/deficiency is expressed as the difference of the observed ratio of immune genes : total genes on each chromosome to the expected random ratio of 1. Width of bars represents the total number of genes on each chromosome expressed as the fraction of *Drosophila* chromosome 3. * indicate significance level from 10′000 randomly distributed sets of immune genes (**: p<0.01, ***: p<0.001).

Immune genes were strongly concentrated on chromosome 2, which displayed a significant excess of immune genes compared to a random distribution (Fig. 1B). The other chromosomes (chromosomes X, 3) were characterised by a significant deficiency in immune genes (Fig. 1B). Within chromosomes immune genes were clustered as well (Fig. 1A). A total of 14 immune gene clusters could be detected in the *Drosophila* genome. Cluster size ranged from 2–15 (mean: 6.33) and each cluster consisted of 1-5 (mean: 3.07) different gene families (Table 1).

**Table 1:** Characteristics of immune clusters in the *Drosophila melanogaster* genome.

Cluster			2.1	2.2	2.3	2.4	2.5	2.6	2.7	2.8	2.9	2.10	3.1	3.2	3.3	3.4
**size**			7	4	15	4	3	2	13	10	4	4	4	7	6	8
**# gene families**			3	2	5	3	2	2	5	5	1	2	2	4	5	3
**Recombination rate [cM/Mb]**			6.9	5	3.4	1.4	1.2	1.1	1.2	1.4	2.3	3.2	3	5	3.5	3.9
**Gene family (group)** 1	**recognition**	*PGRP (11)*	-	-	-	-	-	-	-	3	-	-	-	-	-	-
		*TEP (6)*	-	-	3	1	-	1	-	-	-	-	-	-	-	-
		*GNBP (3)*	-	-	-	-	-	-	-	-	-	-	2	-	-	-
		*SCR (19)*	1	-	3	-	-	-	1	-	-	3	-	-	-	-
		*CTL (32)*	3	2	5	-	-	1	1	-	-	-	-	1	-	-
		*GAL (4)*	3	2	-	-	-	-	-	-	-	-	-	-	-	-
		*FBN (13)*	-	-	-	-	-	-	-	-	-	-	-	1	-	-
	**signalling**	*CLIP (34)*	-	-	-	2	1	-	-	1	-	-	-	3	2	3
		*Srpn (27)*	-	-	3	-	-	-	9	4	-	-	-	2	-	1
		*TOLL (9)*	-	-	-	-	-	-	-	-	-	-	-	-	1	-
		*Cact (1)*	-	-	-	1	-	-	-	-	-	-	-	-	-	-
		*Pel (1)*	-	-	-	-	-	-	-	-	-	-	-	-	1	-
		*SPZ (5)*	-	-	1	-	-	-	-	-	-	1	-	-	1	-
		*TUBE (1)*	-	-	-	-	-	-	-	-	-	-	-	-	-	-
		*REL (3)*	-	-	-	-	2	-	-	-	-	-	-	-	-	-
		*MyD88 (1)*	-	-	-	-	-	-	-	1	-	-	-	-	-	-
		*IMD (1)*	-	-	-	-	-	-	-	-	-	-	-	-	-	-
		*Stat (1)*	-	-	-	-	-	-	-	-	-	-	-	-	-	-
	**effector**	*PPO (3)*	-	-	-	-	-	-	-	1	-	-	-	-	-	-
		*CEC (4)*	-	-	-	-	-	-	-	-	-	-	-	-	-	4
		*AMP(15)*	-	-	-	-	-	-	-	-	4	-	-	-	-	-
		*CASP (7)*	-	-	-	-	-	-	1	-	-	-	-	-	1	-
		*IAP (3)*	-	-	-	-	-	-	-	-	-	-	-	-	-	-
		*PAP (5)*	-	-	-	-	-	-	1	-	-	-	2	-	-	-

1 PGRP = Peptidoglycan recognition protein, TEP = Thioester-containing protein, GNBP = Gram negative binding protein, SCR = scavenger receptor, CTL = C-type lectin, GAL = Galectin, FBN = Fibrinogen, CLIP = Clip containing serine protease, Srpn = Serpins, Cact = Cactus, Pel = Pelle, SPZ = Spaetlzle, REL = relish, PPO = Prophenol oxidase, CEC = Cercropins, AMP = antimicrobial peptides, CASP = caspases, IAP = apoptosis inhibitory proteins, PAP = pre-apoptotic proteins

Numbers in brackets give the number of genes entering the analysis.

### Recombination rates of immune genes and clusters

**Figure 2:**
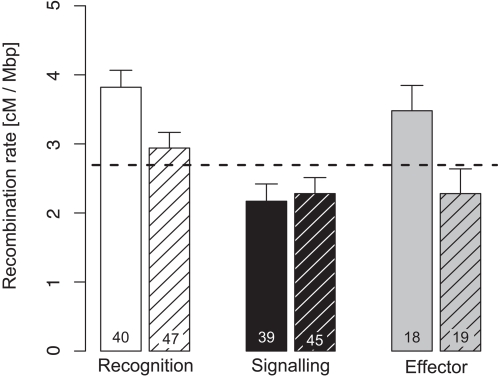
Average local recombination rates (± S.E.) of immune genes in the *D. melanogaster* genome. Immune genes were grouped according to their function during the immune response and whether they were contained in a cluster (open columns) or not (hashed columns). The dashed horizontal line gives the average genomic background recombination rate. The numbers inside each bar represent the number of immune genes found in the respective group. Details on statistical differences can be found in Table 2.

Local recombination rates for different functional classes of immune genes differed significantly from each other and from genomic background for recognition and signalling genes. Recognition and effector genes were found in areas of higher and signalling genes in regions with lower recombination density (Fig. 2). Clustered immune genes lay in regions of significantly higher recombination densities (Table 2). This pattern was mainly driven by those genes interacting with the parasite (i.e. recognition and effector genes), while it was absent for signalling genes (Fig. 2, significant interaction term in Tab. 2). Due to their physical proximity recombination estimates for clustered genes are not independent from each other. To guarantee independence of data points I reanalysed the model by only taking average cluster recombination rates for each functional group contained in the cluster and weighing each average value by the number of genes contained in the respective functional group of each cluster. This conservative approach did not change the pattern observed for the single gene analysis qualitatively (Tab. 2).

**Table 2:** ANOVA table analysing the distribution of local recombination rates according to immune gene function (recognition, signalling, effector) and clustering of the immune genes of *D. melanogaster*.

*Model*	*Factor*	*d.f.*	*F*	*P*
Single gene	Function (F)	2	11.612	**<0.001**
	Clustering (C)	1	7.914	**0.005**
	F×C	2	3.195	**0.043**
	*error*	202		
Cluster average	Function (F)	2	10.557	**<0.001**
	Clustering (C)	1	4.254	**0.041**
	F×C	2	3.220	**0.043**
	*error*	132		

The single gene model considers each gene as an independent data point, while the cluster average only uses average recombination rates of each functional group in a given cluster to control for non-independent data points within each cluster.

The fact that clustered genes were found in regions of high recombination rates might suggest that recombination rate influenced the rate of local gene duplication. There was however no significant correlation between recombination rate and cluster size nor to maximum number of genes from a single gene family per cluster (cluster size: R = 0.12, P = 0.68, max single gene family: R = −0.06, P = 0.84).

### Transcriptional induction

**Figure 3:**
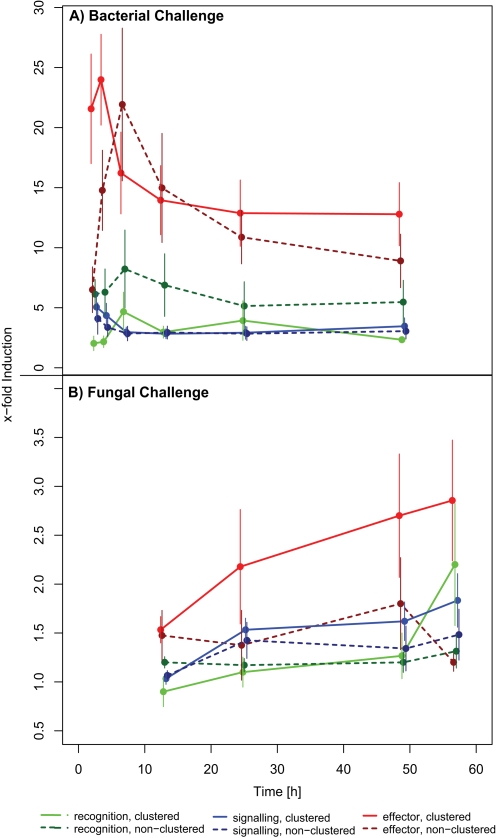
Induction of *D. melanogaster* immune genes after A) bacterial and B) fungal challenge. Expression data was obtained for 50 highly induced or surpressed genes from [25] and genes were grouped according to their function during the immune response and whether they were contained in a cluster (solid lines) or not (dashed lines).

Transcriptional regulation was analysed by comparison of clustered vs. non-clustered genes after septic injury and fungal infection (Table 3 [25]). Effector genes generally reach the highest transcription levels in both types of challenges. During bacterial immune challenges there were strong differences between functional groups as well as between clustered and non-clustered genes within each functional group (Fig. 3A). Clustered effector genes got induced stronger in the beginning of a bacterial challenge whereas non-clustered genes reached their peak during later stages of the infection, probably reflecting the need to facilitate a fast transcriptional response (Kruskal-Wallace test for effector genes at 1.5 h: χ^2^ = 4.321 df = 1, P = 0.038, see also significant Time×Function×Clustering interaction in Tab. 3). Induction of recognition genes showed a different pattern inasmuch that clustered recognition genes displayed lower transcription levels during the whole period.

**Table 3:** Repeated measures MANOVA analysing the effect of immune gene function (recognition, signalling, effector) and clustering on the induction of immune gene transcription during septic injury and fungal infection.

*Induction*		*Factor*	*d.f.*	*F*	*P*
**Bacterial**	between subjects	All between	6,43	6.536	**<0.001**
		Function (F)	2,43	14.903	**<0.001**
		Clustering (C)	1,43	0.051	0.823
		F×C	2,43	1.096	0.343
		Recombination rate (R)	1,43	1.233	0.273
	Within subjects	All within	30,158	1.871	**0.007**
		Time	5,39	2.947	**0.024**
		Time×F	10,78	3.302	**0.001**
		Time×C	5,39	3.216	**0.016**
		Time×F×C	10,78	2.456	**0.013**
		Time×R	5,39	1.292	0.287
**Fungal**	between subjects	All between	6,43	1.941	0.096
		Function (F)	2,43	1.477	0.240
		Clustering (C)	1,43	2.235	0.142
		F×C	2,43	0.699	0.503
		Recombination rate (R)	1,43	0.295	0.590
	Within subjects	All within	18,116	2.017	**0.014**
		Time	3,41	0.306	0.821
		Time×F	6,82	3.676	**0.003**
		Time×C	3,41	4.243	**0.011**
		Time×F×C	6,84	1.077	0.383
		Time×R	3,41	0.306	0.821

Within subjects approximate F values are based on Wilk's λ.

In fungal infections transcription levels were generally lower than during bacterial infetions. During the course of the infection clustered immune genes reach higher transcription levels in all three functional classes, while a strong switch between early and late infection like in the bacterial challenge could not be observed (Fig. 3B). For both types of infection the observed patterns could not be explained by a general correlation between transcription and recombination rate (Tab. 3), indicating that chromatin structure associated with coordinated expression patterns was not leading to more or less recombination events.

### Epistatic interaction between immune gene clusters

Epistatic interactions between immune genes were not distributed randomly in terms of immune gene clusters as well as recombinational distance between interacting genes. The data set of Lazzaro et al. [17] tested 120 pairwise gene interactions. Five out of these pairs could be found within a single cluster, 31 represent pairs between clusters and in the majority of 84 pairs at least one gene did not belong to any cluster. Within these classes the proportion of pairs that showed epistatic interactions differed significantly, with within-cluster and between-cluster epitasis being more common than no-cluster epistasis. In detail 4 out of 5 (80%) within-cluster pairs, 15 out of 31 (48.4%) between-cluster pairs and only 17 out of 84 (20.2%) no-cluster pairs showed epistatic interactions (χ^2^_(d.f. = 2, N = 120)_ = 14.775, P<0.001).

**Figure 4:**
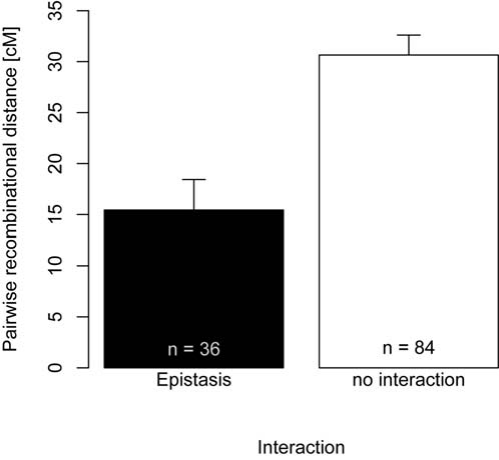
Average recombination distance between of pairs of polymorphic immune genes on chromosome 2 of *D. melanogaster.* Recombination rate (mean±SE) was significantly lower between epistatically interacting genes than between genes that did not show an interaction. Pairs were grouped according to the epistatic interaction between both genes as defined in Lazzaro et. al. [17]. The number of pairs is given in the bottom of each bar.

Recombinational distance between epistatically interacting pairs of was significantly lower than between pairs of genes that did not show such interactions (Fig. 4, F_1,15_ = 18.169, P<0.001, degrees of freedom adjusted to the number of genes). This result was robust against exclusion of no-cluster pairs (F_1,15_ = 18.169, P = 0.004) suggesting that mainly within- and between-cluster epistasis is responsible for the observed pattern.

## Discussion

In this study, I wanted to investigate the spatial and recombinational structure of the *Drosophila melanogaster* immunome and test some alternative hypotheses that could account for the observed clustering of immune genes. Clustering of genes is not unique to genes of the immune systems but the wealth of data available for *Drosophila* immunome in terms of epistatic interactions and expression patterns sets immune genes apart from other functional groups and therefore offers the unique opportunity to potentially discriminate between different evolutionary forces shaping genome architecture. Therefore, I focus on immune genes in this study, where clustering was found between chromosomes with some all chromosomes showing excess or deficiency in immune gene content (Fig. 1B), as well as within chromosomes with regions of significantly higher immune gene density (Fig. 1A). Detection of such clusters strongly depends on the choice of genes entered into the analysis. By using a core immune gene set common to several previous studies I tried to include the most objective set of *D. melanogaster* immune genes. Nevertheless, the formation of functional gene clusters is not necessarily a consequence of selection. A major proportion of genes in the *C. elegans* genome, for exmple, occurs in homologous clusters, which are most likely a consequence of local gene duplications [2]. The high gene family diversity of *Drosophila* clusters could however not be explained by gene duplications alone. While gene duplicaton seemed to cluster size in some effector clusters (e.g. 2.9 and 3.4) other clusters were made up of several different gene families. Cluster composition was nevertheless not random assome clusters consisted of genes with functionally similar background. This became quite obvious for the “recognition clusters” 2.1, 2.2 and 2.3 (Tab. 1) and suggests other selective forces like co-regulation are responsible for cluster formation.

The micro-array data for *D. melanogaster* gene expression after infection with bacteria and fungi [25] showed that there was no significant difference in induction between clustered and non-clustered immune genes in general (Fig. 3, Tab. 2). When incorporating the temporal component of the transcriptional response and functional grouping of genes interesting patterns appeared. Especially the time series of effector genes deserves attention. Here, clustered genes showed a faster response than non-clustered ones (Fig. 3A), which makes sense in terms of a fast response needed against bacterial infection. This pattern was strongly influenced by the anti-microbial peptides (AMP) contained in cluster 2.9, where duplication of AMP genes probably formed a fast inducible effector operon. Non-clustered effectors reached their peak during later stages of the infection. During fungal infection clustered genes of all functional classes increased their expression level relative to non-clustered genes. This lends support the “operon hypothesis” [6] by physically linking genes that are under similar transcriptional regulation [5], [26] and are likely to be found in regions of similar chromatin structure [10]. The transcriptional pattern of recognition genes during bacterial infection, on the other hand, did not lend support to this hypothesis, because clustered recognition genes reach lower transcription levels during the whole immune response. Given the strong degree of clustering of recognition genes in the *D. melanogaster* genome (60.4% of recognition genes occur in clusters compared to 35.7% of signalling genes and 40.6% of effectors, Table 1), other causes might be responsible for clustering of recognition genes.

One feature that both recognition and effector genes share is an increased recombination rate of clustered genes relative to the genomic background (Fig. 2). Such a pattern could not be observed for signalling genes and might reflect different evolutionary forces and constraints working on the different functional classes of immune genes. Gene families of signalling genes show little expansion within the dipterans and mainly consist of orthologous genes with only few paralogs found within each species [27] hinting on evolutionary conservation. Effector and especially recognition genes, on the other hand, belong to gene families that underwent expansion [27], [28] with positive selection acting on recognition genes within the genus *Drosophila* [28] hinting on evolutionary diversification. These two classes are also the major contact points with the parasite. Since recombination rate correlates positively with the likelihood of fixing beneficial mutations in *D. melanogaster* in general [29] the direct interaction between host and parasite molecules might have increased selection for cluster formation in regions of higher recombination rates for these classes of genes (Fig. 2) and thus increased the efficacy of natural selection.

These patterns suggest selection acting on cluster formation but can only tell little about the distribution of clusters on the chromosome. One could hypothesize that the relative position of clusters to each other was a result of epistasis between genes involved in host parasite interactions. The dynamic form of this interaction with changing signs of epistasis [21], [23], [30] might select for higher recombination rates [31]. This might not only have important consequences for the recombination rate around immune gene clusters itself resulting in the formation of recombination hotspots. It might also influence the relative position of immune gene clusters to each other as theory predicts an optimal rather than a maximal recombination rate between epistatically interacting genes [24].

While the genetic architecture of pathogen resistance shows epistatic interactions to a large degree [14] fine scale investigations in closely linked genes are missing. In the studies of Lazzaro et al. [16], [17] a subset of naturally occurring polymorphisms within 16 *Drosophila* chromosome 2 immune genes was tested for epistatic interactions. The distribution of epistatic interaction with respect to clustererd and non-clustered immune genes was not random. The majority of epistatic interactions (19 out of 36) could be found either within a single cluster or between two different clusters. The highest proportion of epistatic interaction was found within clusters (4 out of 5, i.e. 80%). The best covered cluster (i.e. 2.8, Table 1) contains three genetically variable peptidoglycan recognition protein (PGRP), which showed moderate to strong epistatic interactions in infections with four naturally occurring bacteria species [16], [17]. Cluster 2.8 was characterised by a below background recombination rate (Table 1) supporting hypotheses of “supergene” formation, where linkage disequilibrium between positively interacting mutations is only rarely broken up by meiosis and which might explain observations of high LD within *Drosophila* immune receptors [18]. The low recombination rate of this immune cluster along with its widespread epistatic interactions [16] might on the other hand indicate that these particular genes do not experience the co-evolutionary cycles as predicted by the Red-Queen hypothesis. The criterion of high recombination rate was however met by other “recognition clusters” (2.1, 2.2, 2.3). Here, two scavenger receptors (SCR) from 2.2 showed epistatic interactions in two out of the four infection experiments. On the other hand, these SCRs show elevated polymorphism [32] indicating that recombination rate might be linked to polymorphism [33] and could represent a mechanism to generate polymorphism providing the raw material for positive Darwinian selection characterising *Drosophila* immune receptors [28].

Pairs of immune genes that show epistatic interactions could be found at an average recombinational distance of 15.44 cM, which is two times closer together than genes that do not interact (30.64 cM, Fig. 4). The observed average recombinational distance between interacting genes deviated from the optimal theoretical predictions of ∼30 cM [24]. However, this global optimal value might have to be adjusted for the specificities of each species. In this light, the condensed genome of *D. melanogaster* with low overall genomic recombination rates [34], [35] might justify a downscaling of this value and explain the relatively low recombination rate between epistatically interacting genes observed in this genome. Epistatically interacting pairs of genes rarely belonged to the same cluster, which might further indicate that tight packaging of these genes might be associated with selective disadvantages.

In conclusion, it seems that gene regulatory processes [1], [3]–[7] can have a profound effect on cluster formation of *D. melanogaster* immune genes especially in the case of effector genes. On the other hand, epistatic selection for optimal recombination rates [24] that is common between clusters might have arranged the relative positions of cluster to each other. Such selection for linkage disequilibria may advance our understanding of gene order from local gene clusters to the next hierarchical level of chromosomes.

## Materials and Methods

### Genomic resources

For the analysis I used the latest release of the fruit fly genome, *Drosophila melanogaster* (release 5, www.fruitfly.org). Sets of predicted genes were obtained from Flybase (http://flybase.bio.indiana.edu/static_pages/downloads/bulkdata7.html) and all genes were localised in the genome by blasting the coding sequence against the genome sequence. BLAST reports were parsed to identify start and end of each gene. Immune genes were taken from recent comparative studies [27], [36]–[38] focusing on 24 immune gene families groups common to all studies (i.e. leaving out stress related proteins, Tab. 1). This resulted in a total of 207 *Drosophila melanogaster* immune genes (see supplemental material Table S1). Genes were grouped according to their role in the immune response (i.e. recognition, signalling, effector) with grouping criteria derived from descriptions in the databases themselves or relevant publications [27], [36]–[38].

### Identification of immune gene clusters

In eukaryotic genomes genes can be clustered on two different levels. Clustering can occur between chromosomes or within chromosomes. Between chromosome clustering means that certain chromosomes have more immune genes than expected under a random distribution, while within chromosome clustering means that the immune genes found on a chromosome occur in closer proximity than under random expectation. To test for between chromosome clustering, I compared the observed distribution of immune genes to distributions of 10′000 randomised data sets. For each random data set I used the observed distribution of all genes and sampled the original number of immune genes randomly from the whole genome. This way it was possible to calculate the probability to find the observed number of immune genes for each chromosome while still controlling for effects of different gene densities on each chromosome. The excess/deficiency of immune genes was then expressed as differences between the observed immune gene density and the expected gene density under random expectations (Fig. 1B).

For within chromosome clustering a different approach was used. In contrast to other clustering methods, which investigate gene order [39] or homologous clustering [2], I wanted to look at linkage clusters. Physical distance between genes is one of the most important parameters determining linkage. Therefore, I decided to use a sliding window approach, where a window of a size equivalent to the genome-wide average of 2 cM is shifted along chromosomes at an interval of 1 cM. The recombinational distance of 1 cM corresponds to the physical distance of a genomwide average of 0.74 Mb in the *D. melanogaster*. Using a step size of half the actual window size reduces pseudo-replication of genes being contained in multiple bins to a minimum of two bins, but still guarantees the forward and backward connection to the neighbouring bins. Within each window the number of immune genes was counted to obtain a density distribution of immune genes along the chromosomes (black lines in Fig. 1A). Similar to the between chromosome clustering the observed distribution was then compared to 1000 resampled random distributions, where immune genes were randomly distributed along each chromosome according to the observed overall gene densities. For each resample the number of immune genes per bin was counted and the 95% quantile was recorded for each window from all resampled data sets (gray lines in Fig. 1A). An immune gene cluster was then defined for those areas where the observed distribution exceeded this 95% random distribution.

### Recombination rate

To calculate local recombination rates for each gene I used the method of Kliman and Hey (KH93) [40], [41]. This method compares physical maps of genomes with genetic maps by fitting a 4-5 term polynomial functions. This method gives comparative recombination estimates as other such estimates based on physical and genetic maps [40] and has the additional advantage that a local recombination rate can be calculated for every position in the genome by taking the first derivate of the function. Additionally, this method has previously been used for *Drosophila* [29] demonstrating its biological relevance. For calculation of polynomial regression I used the cytotables available at http://flybase.bio.indiana.edu/static_pages/docs/cytotable3.html. Step-wise regressions starting with all five polynomial terms were used to identify the best fitting function describing the relationship between genetical and physical maps. For all chromosomes the functions explained >99% of the variation. Since interactions between hosts and pathogens usually happen on the level of recognition or effector molecules I tested the effect of functional role (recognition, signalling, effector) and clustering (gene in cluster or not) by means of general linear models (GLM).

### Transcriptional induction analysis

To address the hypothesis that immune gene clustering is at least partly caused by co-regulation I looked at the transcriptional response of *Drosophila* to septic injury and fungal infection. The micro-array based genome-wide transcriptional analysis of De Gregorio et al. [25] offers a transcriptional profile over several days. I could identify 50 genes overlapping between the immune gene set used here and the set of those genes that were constantly induced in De Gregorio et al. [25]. Genes were grouped as for recombination rate above, but transcriptional induction was analysed by means of a repeated measures MANOVA with expression level over time as repeated factor.

### Epistatic interactions between immune genes

A manifold of epistatic interactions between naturally occurring polymorphisms of *D. melanogaster* immune genes were previously described for immune genes on chromosome 2 [16], [17]. Since the strength of these interactions varies between changing environments and pathogens [16], I only qualitatively consider the presence of an interaction rather than its quantitative magnitude. The patterns of interactions were analysed with respect to whether interactions were primarily found within or between immune gene clusters as well as recombinational distance between interacting partners.

## Supporting Information

Table S1 List of *D. melanogaster* immune genes
(0.39 MB DOC)
