# Supplementary material for: Clustering of Drosophila melanogaster Immune Genes in Interplay with Recombination Rate
Source: PLoS One. 2008 Jul 30;3(7):e2835. doi: 10.1371/journal.pone.0002835 (PMC2475659; doi:10.1371/journal.pone.0002835)
Supplement: Table S1 — List of D. melanogaster immune genes (0.39 MB DOC) [file pone.0002835.s001.doc]

| Function | Gene family / group ^1^ | Name | LG | Position [bp] | Recombination rate [cM/Mb] | Cluster |
| --- | --- | --- | --- | --- | --- | --- |
| Recognition | PGRP | CG14746-PA | 2 | 26574544 | 1.34E-06 | 2_8 |
|  | PGRP | CG8577-PA | 2 | 26578257 | 1.34E-06 | 2_8 |
|  | PGRP | CG14745-PA | 2 | 26581748 | 1.34E-06 | 2_8 |
|  | PGRP | CG7496-PA | 3 | 7625152 | 3.11E-06 | no |
|  | PGRP | CG4432-PA | 3 | 9316275 | 2.53E-06 | no |
|  | PGRP | CG4437-PA | 3 | 9324835 | 2.52E-06 | no |
|  | PGRP | CG9697-PA | 3 | 16691807 | 9.29E-07 | no |
|  | PGRP | CG9681-PA | 3 | 16692605 | 9.28E-07 | no |
|  | PGRP | CG14704-PA | 3 | 30552549 | 1.08E-06 | no |
|  | PGRP | CG11709-PA | x | 11407498 | 4.10E-06 | no |
|  | PGRP | CG8995-PA | x | 15635550 | 3.43E-06 | no |
|  | TEP | CG7052-PA | 2 | 7700669 | 3.43E-06 | 2_3 |
|  | TEP | CG7068-PA | 2 | 7707966 | 3.43E-06 | 2_3 |
|  | TEP | CG7586-PA | 2 | 8079668 | 3.30E-06 | 2_3 |
|  | TEP | CG18096-PA | 2 | 15889106 | 1.41E-06 | 2_4 |
|  | TEP | CG10363-PA | 2 | 19552021 | 1.08E-06 | 2_6 |
|  | TEP | CG13079-PA | 2 | 19557989 | 1.08E-06 | 2_6 |
|  | GNBP | CG5008-PA | 3 | 8928973 | 2.65E-06 | no |
|  | GNBP | CG4144-PA | 3 | 18638780 | 7.36E-07 | no |
|  | GNBP | CG6895-PA | 3 | 18641554 | 7.35E-07 | no |
|  | SCR | CG4280-PA | 2 | 450540 | 6.91E-06 | 2_1 |
|  | SCR | CG2736-PA | 2 | 42835214 | 5.50E-06 | 2_10 |
|  | SCR | CG2727-PA | 2 | 42838267 | 5.51E-06 | 2_10 |
|  | SCR | CG3829-PA | 2 | 42849860 | 5.51E-06 | 2_10 |
|  | SCR | CG3212-PA | 2 | 3523672 | 5.23E-06 | 2_2 |
|  | SCR | CG3921-PA | 2 | 3796889 | 5.10E-06 | 2_2 |
|  | SCR | CG31962-PA | 2 | 4121195 | 4.94E-06 | 2_2 |
|  | SCR | CG4099-PA | 2 | 4122583 | 4.94E-06 | 2_2 |
|  | SCR | CG12789-PB | 2 | 7446354 | 3.53E-06 | 2_3 |
|  | SCR | CG7228-PA | 2 | 7989177 | 3.33E-06 | 2_3 |
|  | SCR | CG7227-PA | 2 | 7996752 | 3.33E-06 | 2_3 |
|  | SCR | CG2105-PA | 2 | 25422232 | 1.23E-06 | 2_7 |
|  | SCR | CG8856-PA | 2 | 30078338 | 1.86E-06 | no |
|  | SCR | CG4402-PA | 2 | 39650841 | 4.37E-06 | no |
|  | SCR | CG1887-PA | 3 | 1878137 | 5.82E-06 | no |
|  | SCR | CG7422-PA | 3 | 7710045 | 3.08E-06 | no |
|  | SCR | CG10345-PA | 3 | 35697653 | 1.77E-06 | no |
|  | SCR | CG7000-PA | 3 | 40265408 | 2.50E-06 | no |
|  | SCR | CG11335-PA | 3 | 50557974 | 4.08E-06 | no |
|  | CTL | CG13686-PA | 2 | 621136 | 6.81E-06 | 2_1 |
|  | CTL | CG2826-PA | 2 | 625450 | 6.81E-06 | 2_1 |
|  | CTL | CG2839-PA | 2 | 626514 | 6.81E-06 | 2_1 |
|  | CTL | CG3410-PA | 2 | 3717728 | 5.14E-06 | 2_2 |
|  | CTL | CG2958-PA | 2 | 4189224 | 4.91E-06 | 2_2 |
|  | CTL | CG15818-PA | 2 | 7411803 | 3.54E-06 | 2_3 |
|  | CTL | CG6055-PA | 2 | 7472202 | 3.52E-06 | 2_3 |
|  | CTL | CG7106-PA | 2 | 7857875 | 3.38E-06 | 2_3 |
|  | CTL | CG17797-PA | 2 | 8393211 | 3.19E-06 | 2_3 |
|  | CTL | CG17799-PA | 2 | 8394270 | 3.19E-06 | 2_3 |
|  | CTL | CG9976-PA | 2 | 19414015 | 1.09E-06 | 2_6 |
|  | CTL | CG11211-PA | 2 | 24041340 | 1.12E-06 | 2_7 |
|  | CTL | CG14866-PA | 3 | 34416591 | 1.58E-06 | 3_2 |
|  | CTL | CG15358-PA | 2 | 1883186 | 6.09E-06 | no |
|  | CTL | CG15378-PA | 2 | 2035566 | 6.01E-06 | no |
|  | CTL | CG3244-PA | 2 | 4797638 | 4.62E-06 | no |
|  | CTL | CG17011-PA | 2 | 9254831 | 2.90E-06 | no |
|  | CTL | CG16834-PA | 2 | 11002668 | 2.39E-06 | no |
|  | CTL | CG13086-PA | 2 | 19361274 | 1.09E-06 | no |
|  | CTL | CG1652-PA | 2 | 27680484 | 1.48E-06 | no |
|  | CTL | CG1656-PA | 2 | 27682383 | 1.48E-06 | no |
|  | CTL | CG7763-PA | 2 | 29283627 | 1.72E-06 | no |
|  | CTL | CG18431-PA | 2 | 35373465 | 3.07E-06 | no |
|  | CTL | CG14500-PA | 2 | 36056821 | 3.26E-06 | no |
|  | CTL | CG9134-PB | 3 | 1245096 | 6.19E-06 | no |
|  | CTL | CG6014-PA | 3 | 21355407 | 6.02E-07 | no |
|  | CTL | CG4115-PA | 3 | 31461660 | 1.18E-06 | no |
|  | CTL | CG15765-PA | x | 5683425 | 3.60E-06 | no |
|  | CTL | CG12111-PA | x | 8415981 | 4.04E-06 | no |
|  | CTL | CG1500-PA | x | 11828253 | 4.07E-06 | no |
|  | CTL | CG9095-PA | x | 14980392 | 3.59E-06 | no |
|  | GAL | CG11372-PA | 2 | 76095 | 7.14E-06 | 2_1 |
|  | GAL | CG11374-PA | 2 | 77636 | 7.14E-06 | 2_1 |
|  | GAL | CG13950-PA | 2 | 861357 | 6.67E-06 | 2_1 |
|  | GAL | CG5335-PA | 2 | 36489830 | 3.38E-06 | no |
|  | FBN | CG8642-PA | 2 | 26477815 | 1.33E-06 | 2_8 |
|  | FBN | CG10359-PA | 3 | 3597839 | 4.89E-06 | 3_1 |
|  | FBN | CG9593-PA | 3 | 34901935 | 1.65E-06 | 3_2 |
|  | FBN | CG9500-PA | 2 | 6358085 | 3.95E-06 | no |
|  | FBN | CG31832-PA | 2 | 15027649 | 1.54E-06 | no |
|  | FBN | CG17579-PA | 2 | 30668865 | 1.97E-06 | no |
|  | FBN | CG5550-PA | 2 | 34684445 | 2.88E-06 | no |
|  | FBN | CG30280-PA | 2 | 39974392 | 4.48E-06 | no |
|  | FBN | CG30281-PA | 2 | 39975875 | 4.48E-06 | no |
|  | FBN | CG7668-PA | 3 | 19937143 | 6.53E-07 | no |
|  | FBN | CG1791-PA | x | 9809515 | 4.12E-06 | no |
|  | FBN | CG1889-PA | x | 9811618 | 4.12E-06 | no |
|  | FBN | CG6788-PA | x | 17723060 | 2.78E-06 | no |
| Signalling | CLIP | CG18477-PA | 2 | 15658596 | 1.44E-06 | 2_4 |
|  | CLIP | CG4793-PB | 2 | 15920329 | 1.41E-06 | 2_4 |
|  | CLIP | CG6639-PA | 2 | 17451584 | 1.23E-06 | 2_5 |
|  | CLIP | CG8586-PA | 2 | 26558619 | 1.34E-06 | 2_8 |
|  | CLIP | CG8738-PA | 2 | 26562428 | 1.34E-06 | 2_8 |
|  | CLIP | CG8213-PA | 2 | 26836206 | 1.37E-06 | 2_8 |
|  | CLIP | CG8172-PA | 2 | 26872355 | 1.38E-06 | 2_8 |
|  | CLIP | CG3505-PA | 3 | 33757576 | 1.48E-06 | 3_2 |
|  | CLIP | CG4920-PA | 3 | 34428728 | 1.58E-06 | 3_2 |
|  | CLIP | CG5896-PA | 3 | 46258362 | 3.47E-06 | 3_3 |
|  | CLIP | CG5909-PA | 3 | 46261276 | 3.47E-06 | 3_3 |
|  | CLIP | CG9737-PA | 3 | 49341784 | 3.92E-06 | 3_4 |
|  | CLIP | CG9733-PA | 3 | 49344621 | 3.92E-06 | 3_4 |
|  | CLIP | CG11313-PA | 3 | 49829027 | 3.99E-06 | 3_4 |
|  | CLIP | CG18557-PA | 2 | 2845466 | 5.58E-06 | no |
|  | CLIP | CG3117-PA | 2 | 2846939 | 5.57E-06 | no |
|  | CLIP | CG5390-PA | 2 | 10304770 | 2.58E-06 | no |
|  | CLIP | CG9377-PA | 2 | 13360793 | 1.84E-06 | no |
|  | CLIP | CG1299-PA | 3 | 4135290 | 4.62E-06 | no |
|  | CLIP | CG4914-PA | 3 | 14639943 | 1.23E-06 | no |
|  | CLIP | CG4998-PA | 3 | 16305703 | 9.77E-07 | no |
|  | CLIP | CG9372-PA | 3 | 19572503 | 6.73E-07 | no |
|  | CLIP | CG1102-PA | 3 | 23407992 | 5.95E-07 | no |
|  | CLIP | CG3066-PA | 3 | 26861104 | 7.41E-07 | no |
|  | CLIP | CG13318-PA | 3 | 27870380 | 8.16E-07 | no |
|  | CLIP | CG7996-PA | 3 | 32139299 | 1.27E-06 | no |
|  | CLIP | CG4316-PA | 3 | 35239677 | 1.70E-06 | no |
|  | CLIP | CG7432-PA | 3 | 38768240 | 2.25E-06 | no |
|  | CLIP | CG16705-PA | 3 | 42786786 | 2.92E-06 | no |
|  | CLIP | CG2056-PB | x | 8417205 | 4.04E-06 | no |
|  | CLIP | CG2045-PA | x | 9712173 | 4.12E-06 | no |
|  | CLIP | CG6361-PA | x | 18314421 | 2.55E-06 | no |
|  | CLIP | CG6367-PA | x | 18318932 | 2.55E-06 | no |
|  | CLIP | CG15046-PA | x | 18322837 | 2.55E-06 | no |
|  | Srpn | CG6717-PA | 2 | 7582846 | 3.48E-06 | 2_3 |
|  | Srpn | CG7219-PA | 2 | 8005563 | 3.32E-06 | 2_3 |
|  | Srpn | CG8137-PA | 2 | 8241693 | 3.24E-06 | 2_3 |
|  | Srpn | CG14470-PA | 2 | 23785213 | 1.11E-06 | 2_7 |
|  | Srpn | CG9453-PJ | 2 | 24742208 | 1.17E-06 | 2_7 |
|  | Srpn | CG9454-PA | 2 | 24744645 | 1.17E-06 | 2_7 |
|  | Srpn | CG9455-PA | 2 | 24746544 | 1.17E-06 | 2_7 |
|  | Srpn | CG9456-PA | 2 | 24748395 | 1.17E-06 | 2_7 |
|  | Srpn | CG9460-PA | 2 | 24751292 | 1.17E-06 | 2_7 |
|  | Srpn | CG12172-PA | 2 | 25012592 | 1.19E-06 | 2_7 |
|  | Srpn | CG1865-PB | 2 | 25017039 | 1.19E-06 | 2_7 |
|  | Srpn | CG1859-PA | 2 | 25019180 | 1.19E-06 | 2_7 |
|  | Srpn | CG1857-PA | 2 | 25021572 | 1.19E-06 | 2_7 |
|  | Srpn | CG18525-PA | 3 | 34302647 | 1.56E-06 | 3_2 |
|  | Srpn | CG6687-PA | 3 | 34306221 | 1.56E-06 | 3_2 |
|  | Srpn | CG1342-PA | 3 | 49789760 | 3.98E-06 | 3_4 |
|  | Srpn | CG11331-PA | 2 | 6673175 | 3.83E-06 | no |
|  | Srpn | CG4804-PA | 2 | 10063441 | 2.65E-06 | no |
|  | Srpn | CG9334-PA | 2 | 20825499 | 1.05E-06 | no |
|  | Srpn | CG7722-PA | 2 | 28809585 | 1.65E-06 | no |
|  | Srpn | CG10956-PA | 2 | 34990997 | 2.97E-06 | no |
|  | Srpn | CG10913-PA | 2 | 35994271 | 3.24E-06 | no |
|  | Srpn | CG1308-PA | 3 | 4181216 | 4.59E-06 | no |
|  | Srpn | CG3801-PA | 3 | 19016292 | 7.08E-07 | no |
|  | Srpn | CG6680-PA | 3 | 20252222 | 6.38E-07 | no |
|  | Srpn | CG6663-PA | 3 | 20255479 | 6.38E-07 | no |
|  | Srpn | CG12807-PA | 3 | 29097628 | 9.25E-07 | no |
|  | Toll | CG5490-PA | 3 | 45938170 | 3.42E-06 | 3_3 |
|  | Toll | CG18241-PA | 2 | 9089437 | 2.96E-06 | no |
|  | Toll | CG7121-PA | 2 | 13439261 | 1.82E-06 | no |
|  | Toll | CG8595-PA | 2 | 37694736 | 3.74E-06 | no |
|  | Toll | CG8896-PA | 2 | 37979716 | 3.83E-06 | no |
|  | Toll | CG6890-PA | 3 | 15201380 | 1.14E-06 | no |
|  | Toll | CG7250-PA | 3 | 15302484 | 1.12E-06 | no |
|  | Toll | CG5528-PA | 3 | 20296348 | 6.36E-07 | no |
|  | Toll | CG1149-PA | 3 | 26465403 | 7.15E-07 | no |
|  | Cact | CG5848-PD | 2 | 16320956 | 1.35E-06 | 2_4 |
|  | Pel | CG5974-PA | 3 | 46351011 | 3.49E-06 | 3_3 |
|  | SPZ | CG9196-PA | 2 | 42620152 | 5.42E-06 | 2_10 |
|  | SPZ | CG7104-PA | 2 | 7879146 | 3.37E-06 | 2_3 |
|  | SPZ | CG6134-PA | 3 | 46165196 | 3.46E-06 | 3_3 |
|  | SPZ | CG14928-PA | 2 | 11507384 | 2.26E-06 | no |
|  | SPZ | CG9972-PA | 3 | 2869428 | 5.27E-06 | no |
|  | Tube | CG10520-PA | 3 | 23487555 | 5.96E-07 | no |
|  | Rel | CG6794-PA | 2 | 17410261 | 1.23E-06 | 2_5 |
|  | Rel | CG6667-PB | 2 | 17438263 | 1.23E-06 | 2_5 |
|  | Rel | CG11992-PA | 3 | 28143324 | 8.38E-07 | no |
|  | MyD88 | CG2078-PA | 2 | 27172050 | 1.41E-06 | 2_8 |
|  | Imd | CG5576-PA | 2 | 36278580 | 3.32E-06 | no |
|  | Stat | CG4257-PB | 3 | 39639620 | 2.40E-06 | no |
| Effector | PPO | CG8193-PA | 2 | 26908417 | 1.38E-06 | 2_8 |
|  | PPO | CG5779-PA | 2 | 35755911 | 3.17E-06 | no |
|  | PPO | CG2952-PA | 2 | 40925397 | 4.81E-06 | no |
|  | CEC | CG1365-PA | 3 | 49310045 | 3.92E-06 | 3_4 |
|  | CEC | CG1367-PA | 3 | 49311335 | 3.92E-06 | 3_4 |
|  | CEC | CG1878-PA | 3 | 49312576 | 3.92E-06 | 3_4 |
|  | CEC | CG1373-PA | 3 | 49315674 | 3.92E-06 | 3_4 |
|  | AMP | CG4740-PA | 2 | 31263025 | 2.09E-06 | 2_9 |
|  | AMP | CG10816-PA | 2 | 32613821 | 2.38E-06 | 2_9 |
|  | AMP | CG10146-PA | 2 | 32615549 | 2.38E-06 | 2_9 |
|  | AMP | CG18372-PA | 2 | 32617404 | 2.38E-06 | 2_9 |
|  | AMP | CG32279-PA | 3 | 3297907 | 5.04E-06 | 3_1 |
|  | AMP | CG32283-PA | 3 | 3298566 | 5.04E-06 | 3_1 |
|  | AMP | CG10812-PA | 3 | 3300367 | 5.04E-06 | 3_1 |
|  | AMP | CG32274-PA | 3 | 3319114 | 5.03E-06 | 3_1 |
|  | AMP | CG32268-PA | 3 | 3319679 | 5.03E-06 | 3_1 |
|  | AMP | CG10810-PA | 3 | 3353151 | 5.01E-06 | 3_1 |
|  | AMP | CG1385-PA | 2 | 27919073 | 1.51E-06 | no |
|  | AMP | CG8175-PA | 2 | 33276707 | 2.53E-06 | no |
|  | AMP | CG12763-PA | 2 | 36733640 | 3.45E-06 | no |
|  | AMP | CG10794-PA | 2 | 36735426 | 3.45E-06 | no |
|  | AMP | CG7629-PA | 3 | 36724659 | 1.92E-06 | no |
|  | CASP | CG7863-PA | 2 | 23882313 | 1.12E-06 | 2_7 |
|  | CASP | CG7788-PA | 3 | 48898995 | 3.86E-06 | 3_3 |
|  | CASP | CG18188-PA | 2 | 29734375 | 1.80E-06 | no |
|  | CASP | CG5370-PA | 2 | 41412867 | 4.98E-06 | no |
|  | CASP | CG8091-PA | 3 | 9943788 | 2.33E-06 | no |
|  | CASP | CG14902-PA | 3 | 35598860 | 1.75E-06 | no |
|  | CASP | CG7486-PA | x | 492112 | 1.75E-06 | no |
|  | IAP | CG8293-PA | 2 | 33800615 | 2.66E-06 | no |
|  | IAP | CG12284-PC | 3 | 16003934 | 1.02E-06 | no |
|  | IAP | CG12265-PA | 3 | 36249093 | 1.85E-06 | no |
|  | PAP | CG33134-PA | 2 | 24516969 | 1.16E-06 | 2_7 |
|  | PAP | CG8238-PA | 2 | 29556894 | 1.77E-06 | no |
|  | PAP | CG5123-PA | 3 | 18138306 | 7.77E-07 | no |
|  | PAP | CG4345-PA | 3 | 18268945 | 7.66E-07 | no |
|  | PAP | CG6303-PA | 3 | 29421868 | 9.57E-07 | no |

^1^ PGRP = Peptidoglycan recognition protein, TEP = Thioester-containing protein, GNBP = Gram negative binding protein, SCR = scavenger receptor, CTL = C-type lectin, GAL = Galectin, FBN = Fibrinogen, CLIP = Clip containing serine protease, Srpn = Serpins, Cact = Cactus, Pel = Pelle, SPZ = Spaetlzle, REL = relish, PPO = Prophenol oxidase, CEC = Cercropins, DEF = antimicrobial peptides, CASP = caspases, IAP = apoptosis inhibitory proteins, PAP = pre-apoptotic proteins
